# Enhancing Mechanical and Thermal Performance of Recycled PA6/PP Blends: Chain Extension and Carbon Fiber Reinforcement Synergy

**DOI:** 10.3390/ma18051027

**Published:** 2025-02-26

**Authors:** Neslihan Ergun, Mustafa Oksuz, Aysun Ekinci

**Affiliations:** 1 Institute of Graduate Education, Polymer Materials Engineering, Yalova University, Yalova 77200, Turkey; 2 Faculty of Engineering and Architecture, Recep Tayyip Erdogan University, Rize 53100, Turkey; mustafa.oksuz@yalova.edu.tr; 3 Faculty of Engineering, Polymer Materials Engineering, Yalova University, Yalova 77200, Turkey; 4 Polymer Technology Program, Department of Materials and Material Technologies, Yalova Vocational School, Yalova University, Yalova 77100, Turkey; aysun.ekinci@yalova.edu.tr

**Keywords:** recycled polyamide, chain extender, carbon fiber reinforcement, mechanical and thermal properties, composites

## Abstract

To develop novel materials through the recycling of waste polymers and to enhance their mechanical and thermal properties, composites were synthesized using chain extenders (CEs), compatibilizers (PP-g-MA), and short carbon fiber (CF) reinforcements within recycled polyamide 6 (rPA6) and polypropylene (rPP) blends. The recycling of waste polymers holds paramount importance in the context of environmental sustainability. This study investigates the role of additives in effectively improving the properties of recycled polymers. The composites were fabricated using the twin-screw extrusion method and subjected to a comprehensive range of characterizations, including Fourier Transform Infrared Spectroscopy (FTIR), differential scanning calorimetry (DSC), molecular weight analysis, melt flow index (MFI), heat deflection temperature (HDT), tensile testing, impact testing, and Scanning Electron Microscopy (SEM). Additionally, ANOVA statistical methods were applied to analyze HDT, tensile, and impact test results. The findings of this research demonstrate that chain extenders and compatibilizers significantly enhance the mechanical properties of rPA6/rPP blends, while carbon fiber reinforcements markedly improve both tensile strength and impact resistance. Furthermore, the incorporation of rPP led to an approximately 4% reduction in hardness values; however, this loss was effectively compensated by the addition of chain extenders and CF reinforcements, resulting in an overall increase in hardness. It was observed that chain extenders enhanced the elastic modulus and tensile strength by reinforcing interphase bonding, whereas CF reinforcements strengthened the polymer matrix, leading to improved impact resistance. These findings emphasize the synergistic role of chain extenders, compatibilizers, and CF reinforcements in enhancing the mechanical properties of rPA6/rPP blends. The study underscores recycling as both an environmentally beneficial and effective strategy for developing durable, high-performance composites for industrial use. Consequently, the utilization of recycled polymers contributes substantially to the circular and sustainable materials economy, demonstrating the potential for the widespread industrial adoption of such composites.

## 1. Introduction

Plastic consumption plays an important role, especially in the packaging, health, and construction sectors, and this leads to the rapid accumulation of plastic waste [1]. However, it can take decades for these wastes to biodegrade in the natural environment, and traditional storage methods only postpone the environmental problem temporarily [2]. It is predicted that 12 million tons of plastic waste will be buried in landfills by 2050, and the environmental impacts of this situation will reach a level that is difficult to reverse [3]. In this context, reducing the rate of plastic production and effectively recycling existing plastics are of great importance in terms of sustainability. The refunctionalization of polymers and development of renewable plastic resources are critical steps for a sustainable future. Plastic recycling not only reduces the amount of waste, but also minimizes environmental impacts by reducing production costs [4]. Polyamides (PA), in particular, offer significant advantages in recycling processes; however, molecular weight losses during the reprocessing of these materials can lead to the weakening of mechanical properties and reduced thermal stability. This situation can create negative effects such as melting, deformation, and color change, jeopardizing the aesthetic and functional quality of the final products [5]. In addition, the hygroscopic structure of PAs increases their sensitivity to environmental conditions, and this feature becomes more pronounced during the recycling process.

While PA6 has a moisture absorption capacity of 9.5% to 10% of its weight, PA66 has an average water absorption capacity of 8–10% [6,7,8]. PAs, especially PA6 and PA6.6, have a large share in the global market and provide significant economic contribution in various sectors thanks to their wide range of applications. The global PA market has a large sector with an annual production of 8 million tons [2]. This market is expected to grow by 2.2% by 2027, reaching 10.4 million tons and USD 47 billion. This growth rate shows the important role of the PA industry in the industries and the increasing demand. Approximately 67% of the world PA production belongs to PA6 and 33% belongs to PA6.6. The majority of PA6 production is in China, with Asian and Western markets also being important production centers. PA, which has a 30% share in the global engineering plastics market, is expected to reach a value of USD 43.8 billion in 2023 and is expected to grow to USD 55.7 billion by 2028. This highlights the critical role of PAs in the engineering plastics industry [2,3,4,5]. In addition, PAs are widely used in lightweight and fuel-efficient vehicle components. This demonstrates the economic contributions of PAs in the automotive industry and their impact on environmental sustainability. The lightweight structures of plastics create positive effects on the economy by providing fuel efficiency. Improving the recycling processes of PAs will reduce environmental impacts while also providing economic benefits. Developing new strategies for the effective recycling of plastics within the framework of a circular economy will increase environmental sustainability and economic benefits [2,3,9,10,11,12].

In recent years, PAs have become indispensable materials in various industries [13]. This is due to their outstanding mechanical properties such as high modulus [2], impact resistance [14], toughness [15], strength [16], and thermal stability [17]. These properties make PA an ideal and versatile material for demanding applications such as automotive, electronics, and aerospace applications. However, the increasing interest in sustainability and circular economy has made the mechanical recycling of PA increasingly important in the field of engineering thermoplastics [18]. This is especially important in the automotive sector [13], as it supports initiatives to reduce carbon emissions [19], addresses environmental concerns [20], and optimizes the use of raw materials [21]. However, the recycling process also faces a number of challenges. Recycled materials often face difficulties when entering their second, third, or subsequent life cycles. The continuous exposure of plastics to heat, light, and processing conditions can lead to the degradation of the polymer structures, resulting in a reduced impact strength and tensile strength [21]. Additives, such as compatibilizers, chain extenders, and impact modifiers, are needed to transform waste polymers into new materials [22]. Additives in polymer blends, such as crosslinking agents or chain extenders, can modify the rheological and mechanical properties of the material during processing [23,24]. Chain extenders can be low molecular weight monomeric chemicals or polymer materials that increase the molecular weight of the polymer by reacting with the amine or carboxyl end groups of PAs [25,26]. The repeated exposure of PA to thermal, chemical, and mechanical stresses leads to the degradation of the polymer chain structure and a significant decrease in its mechanical properties. This degradation highlights the need for innovative strategies to restore or improve the performance of recycled PA (rPA). An effective method is to blend rPA with other polymeric materials or to add specific additives [22]. At this point, compatibilizing agents [23] and chain extenders [24] play a critical role in enhancing the interfacial adhesion between polymer phases and rebuilding the molecular structure that is disrupted during recycling. These additives strengthen the structural integrity of rPA, allowing the development of materials with properties similar to those of the original PA, thus expanding their applications in engineering fields [25].

**Figure 1:**
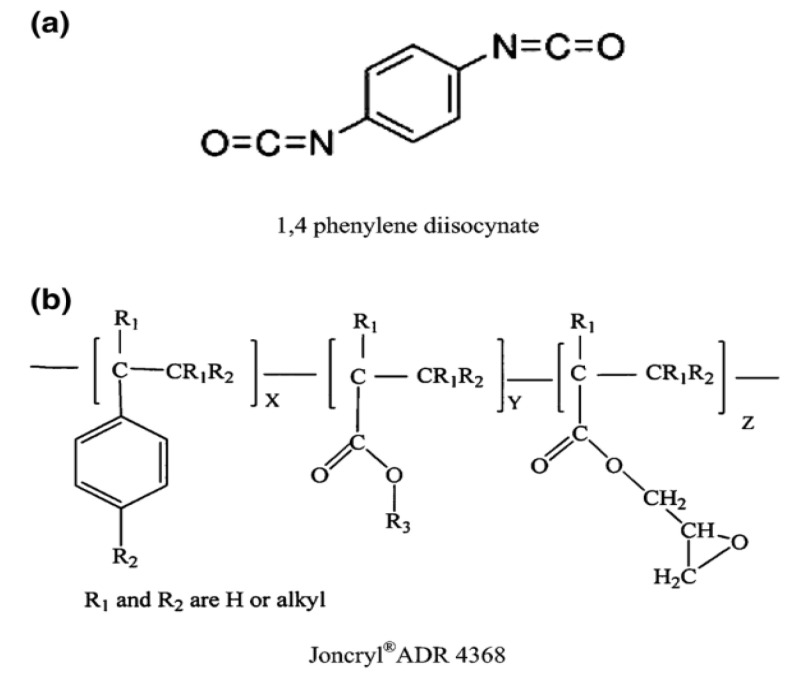
Chemical structures of chain extenders; (**a**) 1,4 phenylene diisocynate, (**b**) Joncryl^®^ ADR 4368 [33].

Polymer blend systems are a widely used cost-effective method to improve the properties of recycled polymer materials [21]. Roeder et al. [27] showed that adding polypropylene (PP) to PA6 composites reduces the material cost and density. The compatibility of polymer blends is a critical factor in determining the physical properties of recycled materials [28]. Incompatible polymer pairs may not exhibit the desired properties in their raw or recycled forms due to differences in their chemical structures and low entropy of mixing [29]. Chain extenders such as diisocyanates, caprolactam, or trimellitic anhydride and polymeric molecules such as epoxy, acrylic acid ester-based monomers, and styrene comonomers (Joncryl) are effective in modifying processing and properties through chain extension [28,29]. Choosing the most suitable chain extender is very important, depending on the processing technology. For injection molding processes, when chain extenders have two or more functions, they cause a partial cure, increasing the elastic properties of the resulting materials [30,31,32]. PAs can be easily modified using monomer mixtures, resulting in copolymers. Additives in PAs are used to improve thermal and photolytic stability and ease of processing, increase flame resistance, provide hydrological resistance, improve friction, and improve the overall properties required for a particular application. Sensitivity to modification is an important factor. Fiber and mineral reinforcements are widely used [28,29]. The compatibility of polymers significantly affects the performance of recycled polymer blends [21], and two or more polymers must be compatible to obtain a homogeneous system [30]. However, some polymers are thermodynamically incompatible, making it difficult to obtain the desired properties by simple mixing processes [31]. To solve this problem, additives such as compatibilizers and chain extenders are used. Chain extenders (CEs), low molecular weight chemicals, react with the amine or carboxyl end groups of PAs to increase their molecular weight [32,33,34]. PP-g-MA has been investigated as a compatibilizer to minimize the tensile strength loss of polymers. CF is often added as a reinforcement to mixtures due to its high tensile strength, hardness, conductivity, and high resistance to temperature, moisture, and corrosive environments. CF has been investigated as a reinforcement in various forms, including short carbon fibers (SCFs) with lengths of 0.5, 1, 2, 5, and 10 mm, long fibers, and bidirectional mats, leading to an increased tensile strength and tensile modulus of PA6 and/or PP composites [32,33,34]. Chemical structures of chain extenders; Figure 1a 1,4 phenylene diisocynate, Figure 1b Joncryl^®^ ADR 4368 are shown in Figure 1 [33].

**Figure 2:**
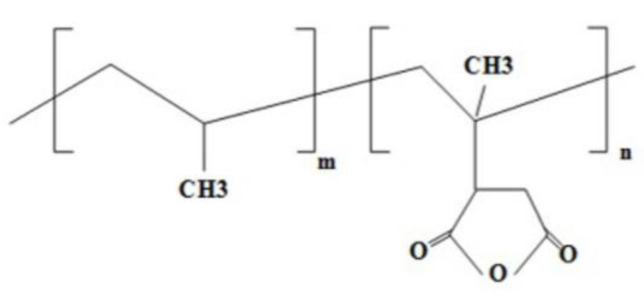
Chemical structure of PP-g-MA [47].

Lozano-Gonzalez et al. [34] observed that although condensation and trans-reactions were achieved, the molecular weight increase due to chain extension could lead to the severe deterioration of nylon upon repeated melting cycles. Studies have shown that virgin PA6 can be maintained for up to seven processing cycles without significantly affecting its physical–mechanical properties or morphology, with the only observed difference being a change in color. After the seventh cycle, the quality declines; however, it has been determined that quality can be improved by blending with virgin material or by adding chain extenders. Various chain extenders have been used effectively in the modification of PAs. Prominent chain extenders include diisocyanate [35], caprolactam [36], trimellitic anhydride [37], and polyepoxides [38]. Costa et al. [39] successfully improved the compatibility of PA6/recycled polyethylene terephthalate (PET) blends using an epoxy-based chain extender and reported that the use of a 1.5% chain extender increased the compatibility. Similarly, Luna et al. [40] observed improved compatibility in PA6 blends modified with maleic anhydride and acrylic acid derivatives, noting that the formation of copolymers increased this compatibility. Ueda et al. [41] investigated carbon fiber (CF)-reinforced PA6/PP blends for water-resistant thermoplastic composites, showing that these blends improved their water resistance. Furthermore, Nguyen-Tran et al. [42] produced CF-reinforced PA6/PP composites with a carbon nanotube (CNT) content to improve mechanical properties and water resistance in automotive applications. Aparna et al. [43] studied the morphology of a PA6/PP (NP) blend under a tensile test and reported that PA6/PP elongated up to 300% with 12% elongation and the fracture was due to the weak bonding between PP and PA6. These studies highlight the critical role of additives and modifiers in improving the physical and mechanical properties of polymer blends. Sridhar and Doddipatla [44] reported that increasing the CF content significantly increased the tensile strength and modulus of PA6/PP composites, but the mechanical properties of the composites decreased with increasing the PP content. Do et al. [45] reported that the mechanical properties of PA6/PP/CF composites decreased with increasing the PP content, but these composites showed potential for application in high humidity or underwater conditions. Aparna et al. [46] observed that the tensile strength increased linearly as the SCF content increased in PA6/PP blends with 3%, 6%, 9%, and 15% SCF content, but the tensile failure rate remained almost constant. It was stated that the impact strength was lower than the samples without SCF. With a 3% and 4% compatibilizer content, small matrix ruptures and the transverse fracture of the matrix were shown as the reasons for composite failures, while at a 5% compatibilizer content, the matrix shear was found to lead to composite failure. The increase in the compatibilizer amount increased the interfacial adhesion between PA6/PP and caused the composites to take on a reticulated appearance. SEM and FTIR results show that there was a transition from brittleness to ductility in the fracture pattern of the composites with the increase in the compatibilizer content. The plasticization of the composites with a high PP-g-MA content (5%) led to the early yielding of the system and a low tensile strength. These findings reveal that the increase in the compatibilizer content in PA6/PP/SCF blends has significant effects on the mechanical performance and significantly changes the fracture behavior of the composites [46]. The chemical structure of PP-g-MA is given in Figure 2 [47].

In this study, recycled polyamide 6 (rPA6) was modified with chain extenders and blended with recycled polypropylene (rPP) to enhance its mechanical and thermal properties. Maleic anhydride-grafted polypropylene (PP-g-MA) was employed as a compatibilizer to improve interfacial adhesion, while the carbon fiber (CF) reinforcement further strengthened the composite. The materials were processed using twin-screw extrusion and injection molding to produce test specimens. A comprehensive characterization, including FTIR, DSC, molecular weight analysis, MFI, HDT, tensile and impact testing, and SEM, was conducted to evaluate the chemical, thermal, mechanical, and morphological properties of the composites. The results indicated that chain extenders significantly enhanced the tensile strength and modulus by improving interphase bonding, whereas the CF reinforcement contributed to substantial increases in both the tensile strength and impact resistance. The synergistic effects of chain extenders, compatibilizers, and CF reinforcements effectively optimized the mechanical performance of rPA6/rPP composites, underscoring their potential for high-performance and sustainable industrial applications.

## 2. Materials and Methods

### 2.1. Used Materials

The rPA6 pellets, which were derived from post-industrial waste originating from automotive interior trim components, were obtained from Komet Chemistry Inc. (Istanbul, Turkey). rPP (recycled polypropylene) was procured from MTM Polymer Inc. (Gaziantep, Turkey). Carbon fiber (CF), based on polyacrylonitrile (PAN) and with a length of 6 mm, was provided by NESA Polymer Inc. (Istanbul, Turkey). The modified styrene/acrylic/epoxy copolymer (CE), commercially known as Joncryl ADR-4368-S, with a molecular weight of 6800 g/mol, an epoxy equivalent weight of 285 g/mol, and epoxy functionality, was sourced from BASF Inc. (Nienburg, Germany). Lastly, PP-g-MA (maleic anhydride-grafted polypropylene), acting as a compatibilizer and commercially identified as COACE B1, was supplied by Zirve Polymer (Istanbul, Turkey).

### 2.2. Sample Preparation

PAN-based CF [48], 6 mm long, was prepared by cutting. Modified styrene/acrylic/epoxy CE, commercially known as Joncryl ADR-4368-S, has a therapeutic weight of 6800 g/mol, an epoxy weight of 285 g/mol, and an epoxy curing agent. Chain extenders are the keys to the polymer chains, which increase the strength of the polymer, and this improves the thermal stability and mechanical properties of biopolymers in particular. Joncryl ADR-4368-S is an important chain extender in this context. This substance, which contains the epoxy group, improves the rheological properties of the polymers, increases the fluidity, and compacts them. The use of chain extenders prevents the mechanical deterioration of the polymers and improves the fluidity during processing, thus providing better processability and durability [49]. Aparna et al. [46] observed that the maintenance of SCF (short carbon fiber) ratios between 3 and 15% in the 70/30 PA6/PP blend was examined and that it increased its shrinkage. Carbon fibers can expand the mechanical strengths and deformations of the polymer matrix, while also improving the thermal resistance of the material. In addition to the degradation of the carbon fiber, the effect of chain extenders improves the sortability of the polymers, while mechanical properties are also added. PAN-based carbon fibers have high strength and low weight properties and have the potential to have mechanical and thermal properties of plastic materials. The chain extenders and carbon fibers used provide greater resistance to the abundance of polymers.

Al-Itry et al. [50] stated that these chain extenders prevented polymer degradation and did not provide weight gain in 1.5% Joncryl distributions in PLA/PBAT blends. However, it was observed that the chain extenders increased the melt stability and resulted in a more stable appearance during the processing process of the material. The use of chain extenders in PLA/PBAT blends has been confirmed by the increase in the viscosity and rheological degradation of the polymers. These changes allow for less thermal degradation and higher performance during polymer modifications. Such modifications allow for stronger, more stable, and environmentally friendly products to be used in the combination of biopolymers and traditional engineering plastics. Additives such as chain extenders and carbon fibers play an important role in the production of sustainable engineering materials, especially those that increase their durability. Frenz et al. [51] emphasized the importance of the recycling capacity of plastics, stating that such modifications can provide contributions to sustainable parts. The need for sustainable conditions is an inevitable reality in the future due to the negative effects of plastic waste. The recycling of non-biodegradable plastics such as polyethylene terephthalate (PET), polycarbonate (PC), and nylon has led to the processing of additives called chain extenders. Chain extenders improve the properties of bioplastics (such as PLA and PHA), allowing them to be used instead of unsustainable polymers and making biopolymers compatible with traditional engineering plastics. The occurrence of these defects improves the processability and mechanical properties of biopolymers, allowing them to be used in more demanding engineering applications. In addition, the low compact weight and limited mechanical properties of bioplastics prevent their use in wider application areas. With these corrections, the effects of chain extenders and carbon fibers on polymers and their logical rationale are more clearly demonstrated. It was emphasized that chain extenders play a critical role in improving the rheological, mechanical, and thermal properties of polymers and making bioplastics compatible with traditional engineering plastics. Carbon fibers have clarified this process by adding strength and durability to the material.

**Figure 3:**
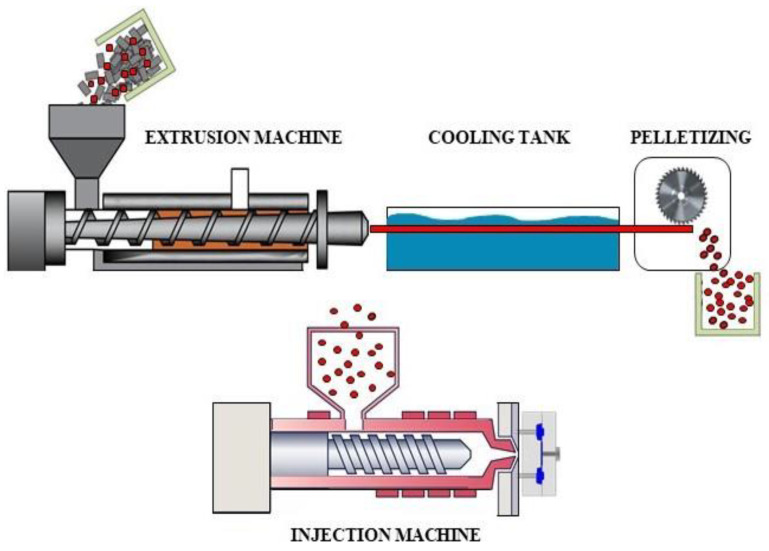
The production scheme for the polymer composites.

The preparation of the composite samples began with drying rPA6 granules in a ventilated oven at 110 °C for 16 h to eliminate any residual moisture. Following this, rPA6, and 0.2%, 0.5%, and 0.75% by weight chain extenders (CEs) were blended in a co-rotating twin-screw extruder (L/D: 40) at 240–270 °C to form a homogeneous mixture. Viscosity tests and melt flow index (MFI) tests were conducted on the CE-PA6 groups to determine the optimal CE concentration for further composite preparation. Viscosity measurements were performed using the Ubbelohde capillary viscometer by the ISO 307 test standard, with formic acid used as the solvent. The MFI test was conducted according to the ISO 1133 test standard, at a temperature of 265 °C and a constant load of 5 kg to determine the flow behavior of the material. To enhance the compatibility between rPA6 and rPP, 2.5% by weight of PP-g-MA was added, and then CF was incorporated into the mixture. The resulting blend was processed in the same co-rotating twin-screw extruder. An additional drying step was applied to remove any remaining moisture before proceeding with the processing stages. The mixture percentages of the components in the composite blends are provided in Table 1, and the production scheme for the polymer composites is given in Figure 3. Once the composites were thoroughly blended, the test samples were molded using an injection molding machine at temperatures ranging from 265 to 280 °C.

### 2.3. Characterization

The presence of functional groups in the composites was determined using a JASCO brand 6600 model Fourier Transform Infrared Spectroscopy (FTIR) analyzer (Tokyo, Japan) in the wavelength range of 400–4400 cm^−1^. Thus, the chemical characterization of all materials was carried out. Differential scanning calorimetry (DSC) analysis was carried out using a TA Instruments DSC250 thermal analyzer (New Castle, DE, USA) at a heating rate of 20 °C/min up to 300 °C and in an inert nitrogen atmosphere. The degree of crystallinity (%) was calculated using the following equation:

$$\mathrm{Xc}=\frac{\Delta Hm}{\Delta H{}^{\circ}m(1-\emptyset )}\times 100$$

In the provided equations, ΔHm is the melting enthalpy of the polymer, ∅ is the polymer fraction in the mixture, and Tm is the melting peak temperature. The specific melting enthalpy (ΔH_m_) of the polymer is compared with the specific melting enthalpy (ΔH_0_) of fully crystalline PA6, which is assumed to be 190 J g^−1^. The mechanical properties of the composites were evaluated by tensile and notched Izod impact tests, as affected by the compatibilizer and carbon fiber content. The tensile test was performed in accordance with ISO 527-2 and ASTM D638 standards, under ambient conditions and at a tensile speed of 50 mm/min. The relative humidity in the laboratory environment was maintained at 48% during the test. The obtained tensile strength values represent the average of five repetitions for each sample. The tensile strength expresses the maximum stress that the material can withstand before breaking. It is calculated with the following formula:

$$\sigma :\;\frac{Fmax}{A0}$$

Fmax = Maximum applied force (N),

A0 = Initial cross-sectional area of the sample (m^2^).

The elastic modulus is the resistance of the material against deformation caused by tension. This is defined as the slope in the linear region of the stress-strain curve and is calculated by the following formula:

$$E:\;\frac{\Delta \sigma }{\Delta \varepsilon }$$

Δσ: Change in stress (N/m^2^),

Δε: Change in elongation rate.

The elongation at break indicates the amount of elongation of the material before it breaks. It is usually expressed as a percentage and is calculated by the following formula:

$$\varepsilon b:\;\frac{\left(Lf-L0\right)}{L0}\times 100$$

Lf = Length at break (m),

L0 = Initial length (m).

Notched Izod impact tests were conducted in accordance with ASTM D256, utilizing a hammer to deliver an impact energy of 5.4 J. The impact strength values for each group were recorded as the average of five samples. The thermal properties of the composite materials were evaluated through Thermal Deflection Temperature (HDT) analysis. HDT measurements were performed using a Instron CEAST HDT 6 Vicat tester (Norwood, MA, USA) under a constant load of 1.80 MPa, following the ISO 75 standard, and the HDT values for each composite formulation were recorded. Hardness tests were conducted using a Shore D durometer, and the average of eight measurements for each group was calculated to enhance accuracy. The thermal stability of the composites was assessed through the High Temperature Deformation Point (HDT) test, as per the ASTM D648 standard. Samples were tested under a load of 0.45 MPa, with the temperature increased from 50 °C to 250 °C. Statistical analysis was performed by calculating the mean and standard deviation for each group based on the obtained HDT values. The statistical significance of the differences among groups was evaluated using a one-way analysis of variance (ANOVA). Tensile tests, conducted in accordance with the ASTM D638 standard using a ZwickRoell Z250 device (Ulm, Germany), provided data on the elastic modulus, tensile strength, and elongation at break. The mechanical and thermal properties, including HDT, elastic modulus, tensile strength, elongation at break, impact strength, and hardness, were systematically analyzed. Standard deviation calculations were performed to ensure data reliability, and statistical significance was determined based on *p*-values (*p* < 0.05), indicating meaningful differences between groups. The impact of the compatibilizer and carbon fiber (CF) content on the morphological properties of the composites was examined using a scanning electron microscope (SEM), ZEISS Supra 40 VP model (Jena, Germany). The fracture surfaces of the samples obtained from the notched Izod impact test were prepared for SEM analysis. Prior to SEM examination, the samples were sputter-coated with a thin layer of gold–palladium (Au-Pd) for 40 s to enhance surface conductivity. SEM images of Group 1, Group 2, Group 3, Group 4, Group 5, and Group 6 samples were captured using a 10 kV voltage setting, ×1.00 kx magnification, Secondary Electron (SE) detector, and a 10 mm working distance.

## 3. Results and Discussion

### 3.1. Structural (FTIR) Analysis

**Figure 4:**
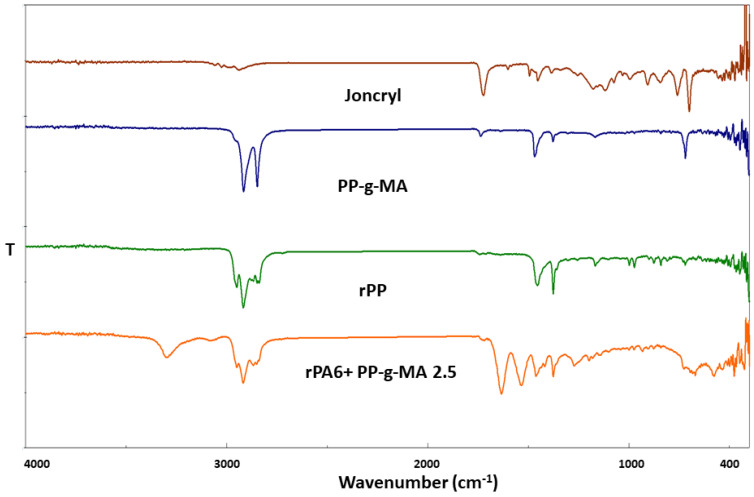
FTIR spectrum of Joncryl, rPP, PP-g-MA, and rPA6+PP-g-MA 2.5 samples.

Joncryl ADR-4368-S, as a chain extender containing epoxy groups, exhibits distinct peaks in the FTIR spectrum. The C–O–C vibration around 910 cm^−1^, C–O stretching vibration at 1200 cm^−1^, and C–H stretching vibration at 2800 cm^−1^ especially confirm the ability of Joncryl ADR-4368-S to enter into chemical interactions by extending polymer chains. The existence of epoxy groups improves the mechanical properties of the material by strengthening the chemical bonds between rPA6 and rPP. PP-g-MA, a polymer modification grafted with maleic anhydride, is characterized by C=O carbonyl stretching vibrations observed at 1780 cm^−1^ and 1850 cm^−1^ in the FTIR spectrum [52,53,54]. These vibrations indicate that PP-g-MA plays a critical role in enhancing the compatibility of the blend by binding maleic anhydride to the PP polymer. Furthermore, C–O bond vibrations reveal that maleic anhydride is effectively grafted onto the polymer surface and helps to reduce phase incompatibilities in rPA6/rPP blends. In the FTIR spectrum of rPP, symmetric and asymmetric CH2 and CH3 group vibrations are evident at 2850 cm^−1^ and 2920 cm^−1^, reflecting the more aliphatic structure of rPP. Since rPP does not possess amide or carboxyl groups, it shows a lower tendency for chemical interactions. However, this limitation can be improved by adding modifiers such as PP-g-MA and Joncryl ADR-4368-S. In the FTIR spectrum of rPA6 + PP-g-MA 2.5 composite, characteristic maleic anhydride peaks containing rPA6 and PP-g-MA blends and epoxy group peaks of Joncryl ADR-4368-S are observed. The presence of epoxy and maleic anhydride groups in the mixture indicates that chemical interactions and compatibility are improved, which strengthens the bonds between rPA6 and rPP phases. This contributes to the increase in mechanical properties by creating a more homogeneous mixture [55,56,57]. FTIR analysis shows that both Joncryl ADR-4368-S and PP-g-MA significantly increase the chemical compatibility between rPA6 and rPP and improve the mechanical and thermal properties of the material. The presence of maleic anhydride and epoxy groups strengthens the chemical bonds between the polymer phases, increasing the durability and performance of the composite. These modifications make the material more efficient and sustainable for industrial applications [56,57,58,59]. FTIR spectra of Joncryl, rPP, PP-g-MA, and rPA6+PP-g-MA 2.5 samples are included in Figure 4.

### 3.2. Thermal (DSC) Analysis

**Figure 5:**
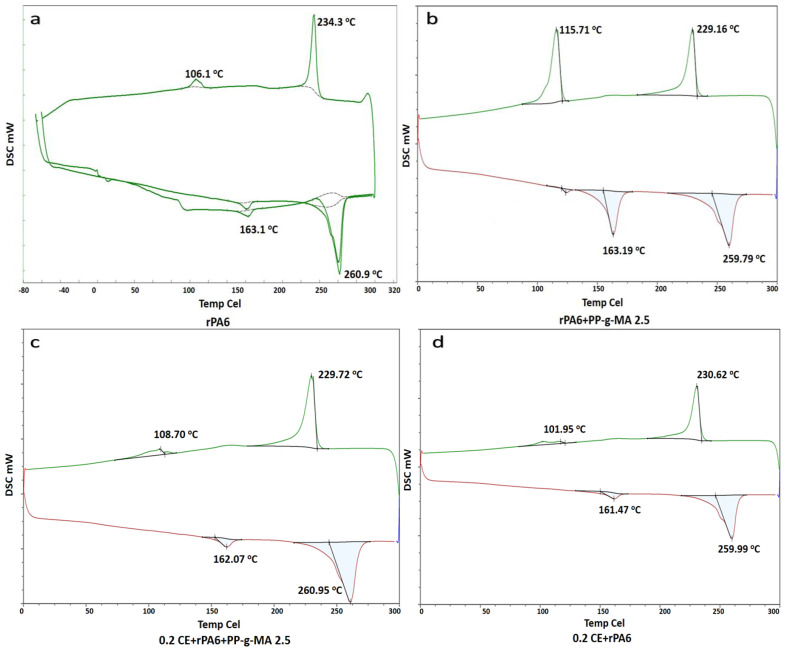

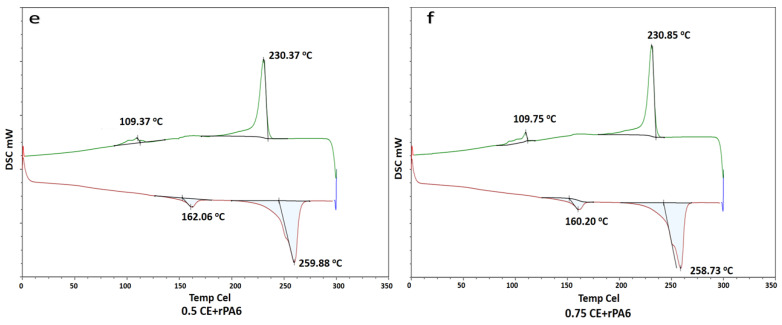
DSC analysis result thermograms of samples (**a**) rPA6, (**b**) rPA6 + PP-g-MA 2.5, (**c**) 0.2CE+rPA6 +PP-g-MA 2.5, (**d**) 0.2CE+rPA6, (**e**) 0.5CE+rPA, and (**f**) 0.75CE+rPA6.

Table 2 shows the DSC analysis results of the composite materials. rPA6 typically exhibits two melting peaks, one at 260.9 °C (higher temperature) and another at 163.1 °C (lower temperature), with the latter reflecting the influence of rPP due to its lower melting point. The crystallization temperatures of rPA6 are observed at 234.3 °C and 106.1 °C, indicating a heterogeneous structure. The addition of PP-g-MA slightly reduced the melting temperature (259.79 °C) and enhanced the second melting peak (163.19 °C), suggesting improved phase compatibility between rPA6 and rPP. The crystallization temperatures decreased slightly and broadened (229.16 °C and 115.71 °C), indicating that PP-g-MA facilitates a more complex crystal structure by promoting interactions between polymer chains [60,61,62,63]. When chain extenders (CEs) were incorporated into the mixture, the melting temperature remained nearly unchanged, but the second melting peak became more distinct (162.07 °C for 0.2CE + rPA6 + PP-g-MA). This highlights improved phase compatibility and a more stable structure due to the extension of polymer chains. Additionally, the crystallization temperatures were slightly lower and more narrowly distributed (229.72 °C and 108.70 °C), suggesting faster crystallization and a more homogeneous structure. As the chain extender concentration increased (0.5CE+rPA6 and 0.75CE+rPA6), the second melting peak further increased, and the crystallization temperatures remained narrowly distributed (230.37 °C and 109.37 °C for 0.5CE, 230.85 °C and 109.75 °C for 0.75CE), demonstrating stronger phase compatibility and a more efficient crystallization process. Overall, the addition of PP-g-MA and chain extenders significantly enhanced the thermal stability, crystallization rate, and phase compatibility of rPA6/rPP blends, promoting a more homogeneous structure with improved long-term performance [64,65]. Thermograms of the samples as a result of DSC analysis for (a) rPA6, (b) rPA6 + PP-g-MA 2.5, (c) 0.2CE+rPA6 +PP-g-MA 2.5, (d) 0.2CE+rPA6, (e) 0.5CE+rPA, and (f) 0.75CE+rPA6 are given in Figure 5.

### 3.3. Molecular Analysis and Reologic Test

Table 3 presents the flow properties of CE/rPA6 blends. It is evident that as the CE concentration increases to 0.2%, 0.5%, and 0.75%, the MFI values decrease consistently, indicating an improvement in melt strength and consequently resulting in enhanced chain extension. This trend has been attributed to the increase in chain extender content, which causes molecular chains to undergo branching during melt flow, leading to an increase in melt viscosity and consequently a rise in molecular weight (Mw) [5]. The viscosity test results were analysed to confirm the observed increase in molecular weight, indicating that the incorporation of a 0.2% chain extender resulted in a higher molecular weight. However, as the chain extender content increased, the molecular weight decreased. Similar findings have been reported in the literature; Baimark and Srihanam [66], as well as Cosate de Andrade et al. [67], highlighted a direct correlation between molecular weight and melt viscosity in their studies, noting that the presence of a chain extender reduces MFI while increasing Mw. In light of these values, the CE concentration to be used in rPA6/rPP blends has been determined to be 0.2%.

### 3.4. Mechanical Thermal (HDT) Analysis

**Figure 6:**
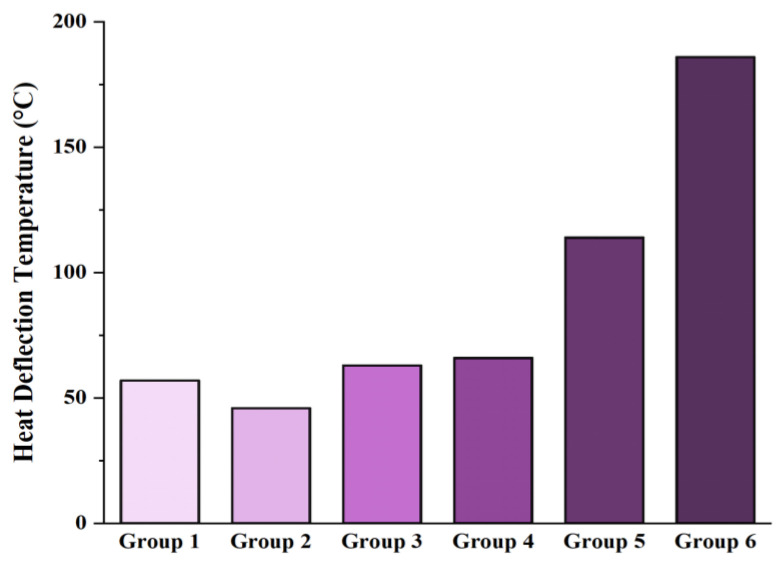
HDT values of rPA6/rPP blend-based composites.

The HDT test results for rPA6/rPP blend-based composites are shown in Figure 6. The measured HDT values were 57 °C for Group 1, 46 °C for Group 2, 63 °C for Group 3, 66 °C for Group 4, 114 °C for Group 5, and 186 °C for Group 6, indicating significant variations in the thermal stability of the composites. A 30% increase in HDT values was observed with the addition of PP-g-MA. A 5% increase in HDT values was achieved with the addition of CE. When 10% CF was added to the CE-based blend, a 42% increase in HDT was achieved. Furthermore, the addition of 20% CF resulted in a 65% increase in HDT. These results demonstrate that the incorporation of carbon fibers and chain extenders significantly enhances the thermal stability of the composites, with a notable improvement in HDT values observed with a higher carbon fiber content. The thermodynamic incompatibility between rPA6 and rPP can negatively affect their thermal properties. Therefore, the incorporation of rPP into rPA6 may weaken the thermal stability and result in a decrease in the HDT value. The literature frequently highlights the beneficial effects of additives, particularly coupling agents and chain extenders, on the thermal properties of polymer blends. These additives can enhance thermal stability by creating stronger bonds between polymer matrices, which can lead to improvements in the HDT values [15,17,68,69,70,71]. The addition of PP-g-MA and CEs has improved the compatibility between the polymers, leading to an increase in the HDT value. This observation is consistent with the findings reported in the literature. CNTs, graphene, fullerenes, and CFs are significant materials that enhance the thermal properties of polymer matrices. The optimal loading of carbon nanofillers can improve the interactions between the polymer matrix and the filler, leading to an increase in thermal degradation temperatures. These interactions reinforce the molecular structure of the polymer, thereby enabling the composite materials to withstand higher temperatures. The incorporation of carbon fibers results in improvements that help maintain the stability of the polymer matrix at elevated temperatures, significantly enhancing the HDT values [72].

The HDT test results of the rPA6/rPP blend-based composites revealed important insights into enhancing thermal stability. Statistical analysis, including ANOVA, showed that the mean HDT value across all groups was 85.17 °C with a standard deviation of 57.68 °C [73,74,75]. The ANOVA test, with an F-statistic of 269.4 and a *p*-value of 1.23 × 10^−^⁶, confirmed that the differences between groups were statistically significant (*p* < 0.05). Notably, increasing the carbon fiber content led to a 42% to 65% improvement in HDT, demonstrating its substantial role in enhancing thermal stability. Additionally, chain extenders (CEs) provided a 5% increase in HDT, with a more pronounced effect when combined with carbon fiber. These findings underline the potential of carbon fiber and CEs to significantly improve the thermal properties of polymer blends, making them promising for industrial applications [74,75,76,77]. HDT values of rPA6/rPP blend-based composites are given in Figure 6.

### 3.5. Mechanical (Tensile) Analysis

**Figure 7:**
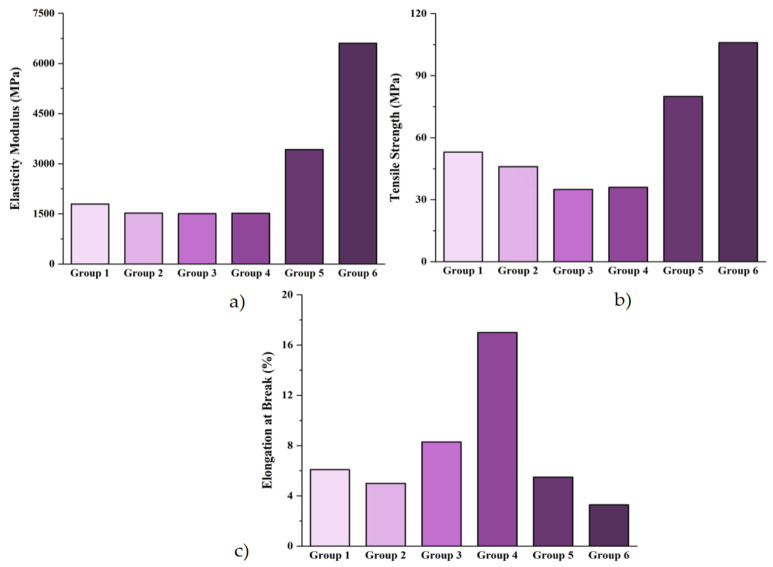
Tensile properties of rPA6/rPP blend-based composites; (**a**) Elasticity Modulus (MPa), (**b**) Tensile Strength (MPa), (**c**) Elongation at Break (%).

Tensile properties of rPA6/rPP blend based composites; (a) Elasticity Modulus (MPa), (b) Tensile Strength (MPa), (c) Elongation at Break (%) are shown in Figure 7. The elastic modulus is considered a measure of a material’s rigidity, as it relates to the material’s resistance to deformation [78]. The analysis of the elastic modulus values revealed the following results: 1796 MPa for Group 1, 1530 MPa for Group 2, 1510 MPa for Group 3, 1520 MPa for Group 4, 3425 MPa for Group 5, and 6606 MPa for Group 6. The addition of rPP into rPA6 resulted in a 14.8% reduction in the elastic modulus. This reduction is attributed to the softer phase introduced by rPP in the rPA6 matrix. The addition of a chain extender increased the elastic modulus. In his study, Tuna [79] related the observed increase in modulus to the concentration of the chain extender. The enhancement was attributed to the increased number of polymer chains being cross-linked, which subsequently raised the viscosity. This effect is linked to a higher molecular weight and chain branching, both of which contribute to improved mechanical properties, including the elastic modulus. With the addition of 10% CF to the polymer matrix, the elastic modulus increased by 125.33%, and with the addition of 20% CF, it further increased by 92.88%. The improvement in hardness with the inclusion of the filler can be explained as follows: Under the influence of a compressive force, the thermoplastic matrix phase and the solid filler phase will press against each other, coming into contact and resisting. This interaction allows for more effective load transfer, even if the interfacial bond is weak. Consequently, the hardness of the filled composites increases [80]. In their study, Hiremath et al. [81] investigated the properties of PAN polymer composites, where they applied a surface treatment to the CF to enhance compatibility with the matrix. The results showed an increase in the elastic modulus with the addition of carbon fibers, attributing this enhancement to the high strength and elastic modulus of the CF. The addition of CF to rPA/rPP polymer composites has enhanced molecular interactions, thereby improving the load transfer efficiency.

According to the tensile strength results presented in Figure 7b, the values for each group are as follows: Group 1 exhibited a tensile strength of 53 MPa, whereas Group 2 showed a reduced value of 46 MPa. For Groups 3 and 4, tensile strength values were recorded as 35 MPa and 36 MPa, respectively. Additionally, higher tensile strength values of 80 MPa and 106 MPa were observed for Groups 5 and 6, respectively. The incorporation of rPP into rPA6 resulted in a 13.21% decrease in tensile strength. This reduction can be attributed to the formation of an incompatible phase structure between rPA6 and rPP and the inherently lower strength of PP. Incompatible phases limit the load transfer due to weak interfacial bonding, thereby reducing the tensile strength of the blend [44]. The inclusion of a 0.2% CE led to an improvement in the tensile strength of polymer blends by approximately 2.86%, indicating enhanced interfacial adhesion and improved phase distribution. Nishida et al. [82] reported in their study that CEs, such as Joncryl, improve phase distribution and enhance the modulus and tensile strength at the break of polymer blends by forming ester bonds. The addition of 10% CF led to a significant increase in the tensile strength at break, showing an improvement of 122.22%. Similarly, with the incorporation of 20% CF, the tensile strength at break increased by 32.5%. These findings highlight the reinforcing effect of CF on the mechanical properties of polymer blends, attributed to the rigid structure and high aspect ratio of CFs, which effectively transfer stress within the composite matrix. Zheng et al. [83] emphasized the significance of chemical bonding within the interfacial enhancement theory to improve the activity and wettability of carbon fibers in their study. They reported that strengthening the interfacial bonds between the reinforcement and the matrix leads to substantial improvements in the mechanical properties of the composite materials. In their study, they also mentioned that epoxy groups are effective in activating the surface of CFs. Additionally, it is suggested that the epoxy groups present in Joncryl may induce a similar effect. The improvement in tensile strength can be attributed to the enhancement at the interface, resulting in a better interaction between the matrix and the reinforcement [84].

Figure 7c presents the elongation at break data obtained during the failure point. Elongation values of 6.1% for Group 1, 5% for Group 2, 8.3% for Group 3, 17% for Group 4, 5.5% for Group 5, and 3.3% for Group 6 were observed. The incorporation of rPP into rPA resulted in a reduction in the elongation capacity of the polymer blend. This can be attributed to the immiscibility and phase separation between rPA and rPP, which typically leads to a less flexible structure, thereby limiting elongation. However, the addition of PP-g-MA resulted in a significant increase of 66% in the elongation, indicating a marked improvement, particularly between Groups 2 and 3. This enhancement is likely due to the reactive interaction between the maleic anhydride groups in PP-g-MA and the rPA matrix, promoting better compatibility and improving the elongation behavior of the blend [31]. The introduction of chain extenders significantly enhanced the polymer’s elasticity and elongation potential, resulting in a notable improvement in the elongation at break. CEs can improve the polymer’s molecular weight distribution, leading to a more flexible structure, thus improving the material’s ability to elongate. In contrast, the addition of CF led to a decrease in elongation, suggesting a detrimental effect of the CF addition on the elongation behavior of the polymer matrix. The stiffening effect of CFs, which act as rigid reinforcements, limits the matrix’s ability to stretch, thereby reducing the elongation at break [85].

Statistical analysis using ANOVA was conducted to assess the effects of rPA6/rPP blend-based composites on mechanical properties, including the elastic modulus, tensile strength, and elongation at break. The average elastic modulus was 3555.17 MPa with a standard deviation (SD) of 2021.89 MPa, indicating significant variability in the data and a heterogeneous structure [86,87,88]. The average tensile strength was 59.33 MPa with an SD of 30.17 MPa, reflecting heterogeneity in the groups’ performance. The elongation at break had a mean value of 7.58%, with an SD of 4.87%, showing significant variation, particularly in the groups containing carbon fiber. The ANOVA results revealed statistically significant differences: for the elastic modulus, the F-value was 5.83 and the *p*-value was 0.005, indicating a significant effect of carbon fiber on the modulus. For the tensile strength, the F-value was 7.25 and the *p*-value was 0.001, confirming that the carbon fiber content significantly enhances tensile strength. For the elongation at break, the F-value was 4.98 with a *p*-value of 0.015, suggesting that the carbon fiber addition limits elongation. These findings highlight the key role of carbon fibers in improving the mechanical properties of rPA6/rPP-based composites, while also demonstrating the beneficial impact of additives like chain extenders on these properties [88,89,90,91].

### 3.6. Mechanical (Izod Impact) Test

**Figure 8:**
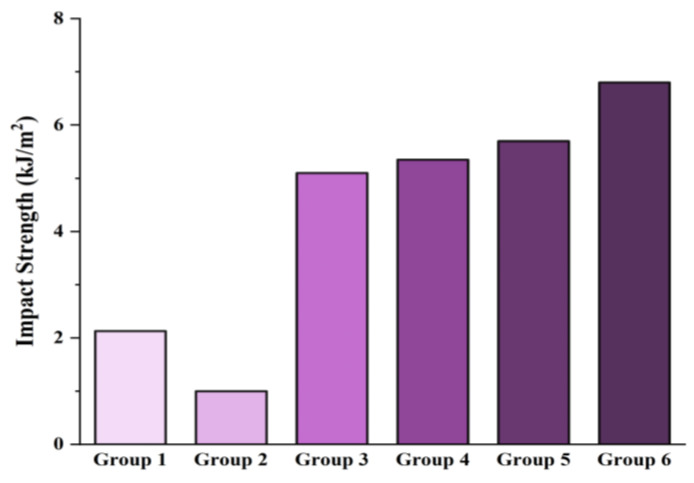
Izod impact strength values of rPA6/rPP blend-based composites.

The impact strength results of rPA6/rPP blend-based composites are presented in Figure 8. The measured impact strength values for the six groups are as follows: Group 1, 2.13 kJ/m^2^; Group 2, 1 kJ/m^2^; Group 3, 5.1 kJ/m^2^; Group 4, 5.35 kJ/m^2^; Group 5, 5.7 kJ/m^2^; and Group 6, 6.8 kJ/m^2^. The rPA6/rPP blends exhibit lower impact strengths compared to the neat rPA6 matrix. This reduction can be attributed to the thermodynamic incompatibility between rPA6 and rPP polymers. The literature indicates that such incompatibility in polymer blends leads to phase separation within the material, thereby adversely affecting mechanical performance. Consequently, the rPP phase is unable to effectively absorb fracture energy, resulting in a significant decline in impact strength [92]. However, the addition of a compatibilizer such as PP-g-MA to the rPA6/rPP blends has resulted in a significant improvement in impact strength. PP-g-MA enhances interfacial adhesion between rPA6 and rPP, thereby improving interphase compatibility and subsequently increasing the mechanical performance of the blend. This observation is consistent with findings in the literature, where the incorporation of compatibilizers has been frequently reported to strengthen interfacial interactions in polymer blends, leading to enhanced impact strength [93,94]. The addition of a CE and CF to PA6 has significantly improved its impact strength. Chain extenders increase the molecular weight of the polymer matrix, which not only enhances its energy absorption capacity but also contributes to reducing crack formation by improving the overall structural integrity of the material [95]. Carbon fibers facilitate effective load transfer during impact, distribute stress, and delay crack propagation. Wang et al. [96], in their study investigating the effects of CF on PP, suggest that there is a strong interfacial bond between the matrix and fibers, which increases the impact toughness.

Statistical analysis using ANOVA was performed to evaluate the impact resistance of rPA6/rPP blend-based composites. The F-value was 8.75, and the *p*-value was 0.002, indicating statistically significant differences in impact resistance between the groups [97,98,99]. Standard deviations were calculated for each group: Group 1 (2.13 kJ/m^2^, SD = 0.15), Group 2 (1 kJ/m^2^, SD = 0.10), Group 3 (5.1 kJ/m^2^, SD = 0.25), Group 4 (5.35 kJ/m^2^, SD = 0.30), Group 5 (5.7 kJ/m^2^, SD = 0.20), and Group 6 (6.8 kJ/m^2^, SD = 0.18). The significant differences highlight the positive effect of carbon fiber (CF) and chain extenders (CEs) in enhancing impact resistance, demonstrating their role in improving energy absorption and preventing crack propagation. These findings suggest that CF and CE additives contribute to the improved mechanical performance of polymer composites, making them suitable for industrial applications [98,99,100].

### 3.7. Hardness Test

**Figure 9:**
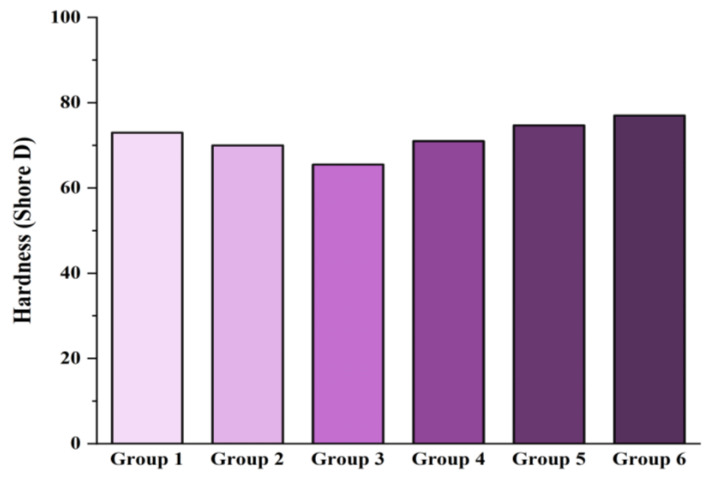
Hardness values of rPA6/rPP blend-based composites.

The hardness values of the rPA6/rPP blend-based composites are presented in Figure 9. The experimental results for Groups 1 through 6 are as follows: 73, 70, 65.5, 71, 74.7, and 77 Shore D. These results indicate a decrease of approximately 4% in hardness with the addition of rPP into rPA. Savas et al. [101], in their studies investigating the effect of bone ash on PA6/PP blends, reported that increasing the PP content led to a decrease in hardness. Although PA6 is a high strength polymer, its ductility is relatively lower compared to PP. Moreover, the addition of PP-g-MA resulted in a decrease in hardness by approximately 6%. In contrast, composites reinforced with chain extenders showed an increase in hardness. This suggests that the CEs enhance the polymer’s structure, leading to higher hardness values. Furthermore, incorporating CF into the polymer composites resulted in continued hardness improvement. CF, known for its high strength and rigidity, positively influences the mechanical properties of the blend. In the literature, previous studies generally report an increase in hardness values due to the high surface area and hardness characteristics inherent to carbon-based nanomaterials. Additionally, the use of coupling agents is believed to enhance the chemical compatibility between the NH groups in the nylon structure and the CFs, promoting covalent and hydrogen bonding interactions [102,103]. These interactions contribute to the improvement of the mechanical properties of the composite materials, further supporting the observed increase in hardness. Furthermore, the relationship between the elastic modulus and hardness has been widely acknowledged in the literature, and the results of the elastic modulus and hardness obtained in this study show consistent and parallel trends, further validating the findings [104].

Statistical analyses of the hardness values of rPA6/rPP blend-based composites were performed using ANOVA to assess significant differences between the groups. The F-value was 9.56, and the *p*-value was 0.003, indicating statistically significant differences in hardness [105,106,107]. Standard deviations for each group were as follows: Group 1: 73 Shore D (SD = 1.20), Group 2: 70 Shore D (SD = 1.15), Group 3: 65.5 Shore D (SD = 1.10), Group 4: 71 Shore D (SD = 0.90), Group 5: 74.7 Shore D (SD = 0.85), and Group 6: 77 Shore D (SD = 1.30). The results demonstrate that carbon fiber (CF) and chain extenders (CEs) significantly improve the hardness of the composites, enhancing their structural integrity. In contrast, the decrease in hardness with the addition of PP-g-MA suggests phase incompatibility. These findings highlight the potential of CF and CEs to optimize the mechanical performance of the composites [106,107,108].

### 3.8. Morphological (SEM) Analysis

**Figure 10:**
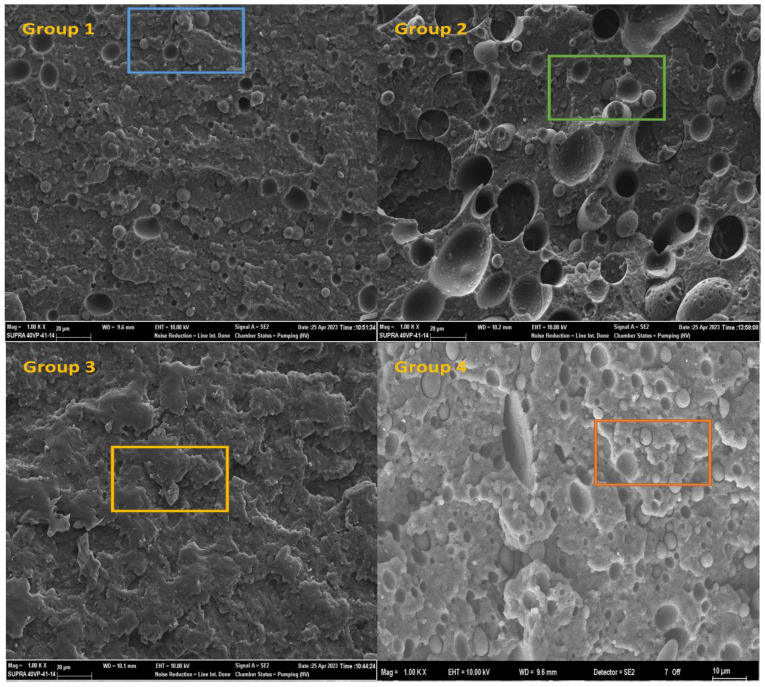

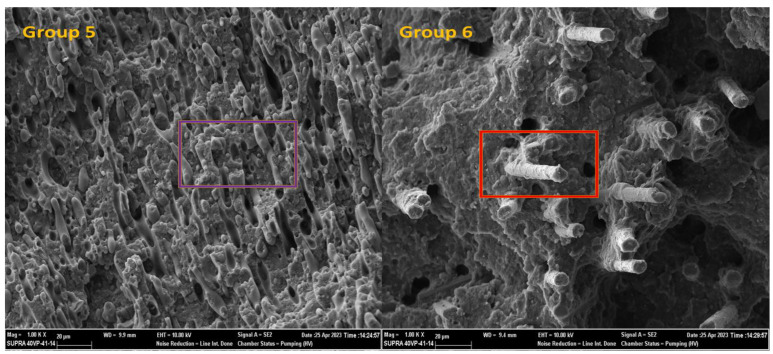
SEM images of Group 1, Group 2, Group 3, Group 4, Group 5, and Group 6 samples (10 kV voltage rate, ×1.00 kx magnification ratio, SE (Secondary Electron) detector, 10 mm working distance). There are differences in material morphology with different color squares.

The SEM micrograph of rPA6 reveals the presence of small particles in the matrix, which is associated with the incorporation of rPP. DSC analysis further confirms this observation by indicating the emergence of a secondary melting peak, confirming the presence of rPP in the matrix material. This phenomenon is consistent with the automotive industry using recycled materials in rPA6. The blue marked region in the SEM micrograph provides a detailed view of these rPP particles in the matrix, supporting their presence. The addition of rPP to rPA6 further emphasizes the visibility of these particles. In the SEM micrograph of Group 2, the weak interfacial adhesion due to the phase mismatch of rPP is clearly visible. This weak interface causes the separation of rPP from the matrix and the formation of distinct voids within the structure, as highlighted in the green marked regions. These observations are in agreement with the recorded decrease in impact strength. The obtained micrographs are consistent with those reported in the literature for PA6/PP blends [61]. However, the incorporation of PP-g-MA significantly increases the dispersion of rPP within the rPA6 phase. This improvement contributes to better matrix-phase compatibility, resulting in a more homogeneous structure. In SEM micrographs of CF-reinforced polymer composites, the fiber surfaces appear to be coated with resin, indicating robust fiber/matrix interfacial adhesion. The red-marked regions in these micrographs provide a detailed view of the fiber surfaces. Such strong interfacial bonding confirms the observed improvements in mechanical properties. These findings are consistent with previously reported results in the literature [45]. SEM images of Group 1, Group 2, Group 3, Group 4, Group 5, and Group 6 samples (10 kV voltage rate, ×1.00 kx magnification ratio, SE (Secondary Electron) detector, 10 mm working distance) are included in Figure 10.

## 4. Conclusions

The study investigated the improvement of mechanical and thermal properties in rPA6/rPP blends using chain extenders (CEs), compatibilizers (PP-g-MA), and carbon fibers (CFs). The chemical interactions between rPA6 and rPP highlight the role of maleic anhydride and epoxy groups in improving the compatibility. The addition of chain extenders and compatibilizers improved the tensile strength and elongation at break, resulting in a more homogeneous blend. Chain extenders improved the melt strength and viscosity, improving thermal stability and processability, thus optimizing crystallization. Carbon fibers significantly improved the impact resistance by reinforcing the polymer matrix. While rPP reduced the stiffness, chain extenders and CF reinforcements compensated for this by increasing the stiffness and flexibility. Statistical tests (ANOVA) confirmed that chain extenders and carbon fibers played important roles in the improved mechanical and thermal properties. The inclusion of CEs, PP-g-MA, and CF significantly enhanced the performance of rPA6/rPP blends. These findings support the potential of using recycled polymers for high-performance composites, advancing sustainable materials design. Future studies should investigate a wider range of additives and evaluate the impact of environmental factors on composite performance.

## Figures and Tables

**Table 1:** Formulations and component ratios of the samples (% weight).

Sample	rPA6				CE
rPA6	100				-
0.2CE-rPA6	99.8				0.2
0.5CE-rPA6	99.5				0.5
0.75CE-rPA6	99.25				0.75
	**rPA6**	**CE**	**rPP**	**PP-g-MA**	**CF**
Group 1	100	-	-	-	-
Group 2	70	-	30	-	-
Group 3	67.5	-	30	2.5	-
Group 4	67.5 (99.8 + 0.2)		30	2.5	-
Group 5	90 (67.5 + 30 + 2.5)				10
Group 6	80 (67.5 + 30 + 2.5)				20

**Table 2:** DSC analysis result thermogram values of the samples (**a**) rPA6, (**b**) rPA6 + PP-g-MA 2.5, (**c**) 0.2CE+rPA6 +PP-g-MA 2.5, (**d**) 0.2CE+rPA6, (**e**) 0.5CE+rPA, and (**f**) 0.75CE+rPA6.

Sample	Tm (°C)	Tc (°C)
(a) rPA	260.9/163.1	234.3/106.1
(b) rPA6 + PP-g-MA 2.5	259.79/163.19	229.16/115.71
(c) 0.2CE+rPA6 +PP-g-MA 2.5	260.95/162.07	229.72/108.70
(d) 0.2CE+rPA6	259.99/161.47	230.62/101.95
(e) 0.5CE+rPA6	259.88/162.06	230.37/109.37
(f) 0.75CE+rPA6	258.73/160.20	230.85/109.75

**Table 3:** Flow properties of CE/rPA6 blends.

Sample/Parameters	Mw (g/mol)	MFI (g/10 min)
rPA6	11.5	102.9
0.2CE+rPA6	22.3	30.1
0.5CE+rPA6	11.0	22.4
0.75CE+rPA6	7.5	7.7

## Data Availability

The data supporting the findings of this study are available from the corresponding author upon reasonable request. However, the data are not publicly available due to confidentiality considerations related to the patent application process. The patent and its associated intellectual property originate from our team during the preparation and application phase.

## Abbreviations

The following abbreviations are used in this manuscript:

CE	Chain extender
CF	Carbon fiber
PA	Polyamide
DSC	Differential scanning calorimetry
HDT	Heat deflection temperature
PP	Polypropylene
